# Knowledge and use of unauthorized HIV self-test kits among men who have sex with men in Spain, following approval of an over-the-counter self-test in the U.S: a cross-sectional study

**DOI:** 10.1186/s12889-016-3204-9

**Published:** 2016-07-08

**Authors:** Konstantinos Koutentakis, María Elena Rosales-Statkus, Juan Hoyos, Sonia Fernández-Balbuena, Mónica Ruiz, Cristina Agustí, Luis de la Fuente, María José Belza, the Madrid HIV self-testing group

**Affiliations:** European Programme for Intervention Epidemiology Training (EPIET), European Centre for Disease Prevention and Control (ECDC), Stockholm, Sweden; National Epidemiology Centre, Carlos III Health Institute, Madrid, Spain; Consortium for Biomedical Research in Epidemiology & Public Health (CIBERESP), ᅟMadrid, Spain; National School of Health, Carlos III Health Institute, Madrid, Spain; Centre for Epidemiological Studies on STI/HIV/AIDS in Catalonia (CEEISCAT)-ICO-Agència de Salut Pública de Catalunya, Barcelona, Spain

**Keywords:** HIV testing, Preventive behaviour, Men who have sex with men, Self testing

## Abstract

**Background:**

Shortly after the approval of an over-the-counter HIV self-test in the US, we conducted a study to estimate the proportion of men who have sex with men (MSM) in Spain who knew that unauthorized HIV self-tests could be purchased online, and the proportion that had already used these tests, as well as their socio-demographic and behavioural correlates.

**Methods:**

Between September 2012 and February 2013, MSM users of gay dating websites were invited to complete an online questionnaire. We calculated estimates of the knowledge and use of unauthorized HIV self-testing and assessed the associated factors by rare event logit regression models.

**Results:**

Among 8620 participants, 4.2 % (95 % CI:3.8–4.6) knew they could buy an unauthorized HIV self-test kit online, and 12.7 % (95 % CI:12.0–13.4) thought that such a test might exist, although they had never seen one. Only 0.7 % (95 % CI:0.5–0.9) had ever self-tested. In the multivariable analysis, knowledge of online availability of self-tests was associated with being a non-Latin American foreigner, having at least two previous HIV tests, intending to test for HIV in the next year, and knowing about U.S. approval of self-testing. Ever-use of HIV self-testing was associated with being over 34 years of age, living outside Spain during the last 12 months, and knowing about U.S. approval of self-testing.

**Conclusions:**

Both knowledge and use of unauthorized HIV self-testing among MSM in Spain was very low among HIV negative or untested MSM in Spain. The recent approval in the United Kingdom and France might increase the number of MSM seeking such testing and possibly using unauthorized test kits not meeting quality standards.

**Electronic supplementary material:**

The online version of this article (doi:10.1186/s12889-016-3204-9) contains supplementary material, which is available to authorized users.

## Background

Rapid test kits to determine the status for HIV and other sexually transmitted infections (STI) have been sold online for years [1]. However, the results of a study conducted to assess the use of internet sites for STI testing and to obtain information about the services offered and validity of the tests were not promising. The internet sites offering HIV tests were found to be difficult to contact, and they were often unwilling to answer consumer-specific questions. Furthermore, the validity of the tests was far from optimal [2].

The approval in 2012 of an over-the-counter HIV self-test for lay people by the United States Food and Drug Administration (FDA) [3, 4] was a crucial event for this testing option in particular, and for HIV testing approaches in general [5]. Its approval was followed by great media coverage, which paved the way for the popularization and future expansion of this testing option, not only in the U.S., but also in Europe.

Thus, in October 2013, the European parliament approved a proposal that left to member states the decision on whether to allow or restrict HIV self-testing. Since then, the United Kingdom (UK) [6] and France [7] have approved and are currently selling HIV self-testing kits (two versions of Chembio's SURE CHECK® HIV 1/2 testing device under the brand name “BioSURE HIV self test” in the UK and “Autotest VIH” in France) [8, 9]. In Spain and the rest of Europe the sale of self-tests remains unauthorized.

Between 2010 and shortly before U.S. approval of HIV self-testing, we performed the first study in Spain to estimate the percentage of potential users who knew that unauthorized HIV self-tests could be purchased online and the percentage that had already used them among people receiving HIV rapid testing at a street-based program [10]. Given its study population, that study was unable to collect information on people who had never been tested (those who responded not having tested before were de facto receiving their first one that day).

Immediately after the approval of HIV self-testing in the U.S. and the mass media attention that followed, we initiated a new study focusing on men who have sex with men (MSM) – the group most affected by HIV in our country – to capture information on individuals with no previous testing experience. MSM non-testers are one of the population groups that could benefit the most from approval of HIV self-testing, given their high vulnerability and reluctance to attend existing sites [11, 12].

In the context of the media exposure that followed U.S. approval of HIV self-testing, we calculated the proportion of MSM in Spain who knew that unauthorized HIV self-tests could be purchased online, as well as the proportion who had already used such tests, and estimated the socio-demographic and behavioural correlates associated with both knowledge and use.

## Methods

### Study procedures and participants

The study was conducted online from September 2012 to February 2013 via dating and chatting websites oriented to gay men, as well as via organizations of lesbian, gay, transsexual and bisexual (LGTB) individuals and non-governmental organization (NGO) sites. On the websites’ front page display, links or banner advertisements invited users to take part in the survey. Some associations also emailed their members inviting them to participate. A total of 68 % of the respondents were recruited from three participating internet sites.

Those who chose to access the survey website were informed of the rationale and purposes of the study. Participants were also informed of the estimated time to complete the questionnaire (15 min). No Internet Protocols or cookies were collected. The questions were designed to elicit no identifying information; for example, participants were asked to report their age in years rather than their date of birth. Likewise, we did not include questions on the city or town of residence; instead, we asked only about the approximate number of inhabitants. As informed consent was not possible, candidates were asked to click on an “I agree to participate button” before having access to the questionnaire. Participants were able to go back to previously answered questions, in order to check and/or change them, before final submission. The eligibility criteria were: a) age ≥18 years, b) being male at birth and ever having had sex with a male and c) currently residing in Spain and accessing the survey page while in the country. The study was approved by the Carlos III health institute ethical committee (CEI PI 70_2015).

### Data collection instrument

We developed an online self-administered questionnaire which covered demographic characteristics, sexual orientation and the involvement with the gay scene. Involvement was assessed with a 4 item question: “I am a member of a LGTB Community Based Organization” “I attend gay venues with my friends on a frequent basis” “I only attend gay venues to hook up with men” “I have virtually never attended a gay venue”. Participants answered “yes” or “no” to each one of them. Those who answered "yes" to the first item were labeled as "Member of a gay CBO" regardless of the other items. Those who answered "no" to the first item but answered "yes" to the 2nd or 3rd item, were labeled as "Not member of a gay CBO-Frequents the gay scene". Finally those who answered "yes" to the 4th item were labelled as "No involvement with gay scene".

Questionnaire also assessed the number of sexual relationships in the last 12 months, risk behaviours, HIV/STDs status, and HIV testing history. Those participants who self-reported being HIV positive were asked about the process leading to their positive diagnosis, possible missed opportunities for earlier diagnosis, and the process of linkage to care. For those who had never been tested or who self-reported being HIV negative – i.e., the future potential users of self-testing – we inquired about self-testing. First, their knowledge of the possibility of purchasing an unauthorized HIV self-test online was measured by a question with three response options: “Yes, I knew about it”, “I thought it could exist, although I had never seen it” and “I had no idea about it”. Those who knew about self-testing were asked if they had ever self-tested. They were asked to choose one of the three following responses: “Never”, “Yes, once”, and “Yes, more than once”. We also included a specific section for those who reported having ever performed a self-test. It included questions on the exact number of self-tests conducted and on the difficulty to follow the instructions of each of the steps of the testing process, which was assessed with a five-item scale question: 1 = Very easy, 2 = Quite easy, 3 = Somewhat difficult, 4 = Quite difficult, 5 = Very difficult. The dataset supporting the conclusions of this article is included within the article and its Additional file 1.

### Data analysis

In total, 13,150 users submitted a completed questionnaire, of whom 9489 self-identified themselves as HIV negative or had an unknown status and therefore were given the opportunity to answer the questions related to self-testing. We excluded 55 participants who stated they were under 18 years old or had some inconsistent responses regarding their HIV status, and 814 subjects were also excluded for not answering the questions related to HIV self-testing. The final sample used for the analysis was 8620 participants.

We calculated proportions for the qualitative variables and, to summarize the quantitative variables, we grouped them into categories and calculated the relative frequencies. 5-point scale variables were treated as categorical. We performed a descriptive analysis of the categorical characteristics stratified by HIV testing history (ever tested/never tested), and we calculated the chi-square statistic to test for dependency between them. Some categories of the independent variables were merged to allow comparisons in statistical testing. The two main outcome variables were re-coded into binary ones in order to build logit regression models. The first dependent variable, which examined the proportion of MSM that knew about HIV self-testing, was grouped as “Yes I knew about it” and “I thought it could exist, although I had never seen it/I had no idea about it”. Similarly, the second dependent variable, which measured the proportion of HIV self-testers, was re-coded as “Ever self-tested, once or more times” and “Never self- tested”.

Estimations of factors associated with the knowledge and ever-use of unauthorized HIV self-testing were examined by calculating odds ratios (OR) and the corresponding 95 % confidence intervals (CI) through logistic regression for rare events data [13, 14]. All the statistically significant factors at *p* ≤ 0.1 in the single variable analysis were entered in a multivariable rare event logit regression model that was built for each outcome. All these analyses were performed using STATA v12 software using the relogit command.

## Results

### Demographic characteristics

A detailed descriptive analysis of the characteristics of the sample stratified by HIV testing history is presented in Table 1, which shows statically significant differences in the distribution of several variables between those who have never been tested (28.6 %) and those with testing experience (71.4 %).

**Table 1:** Sociodemographics, risk behaviors and HIV testing intentions by HIV testing history of MSM websites' users

	Never tested *n* = 2464 (28.6 %)		Ever tested *n* = 6156 (71.4 %)		Total *n* = 8620		
	*n*	%	*n*	%	*n*	%	*P*-value^a^
Age							<0.001
18–24	852	34.6	815	13.3	1667	19.4	
25–34	836	34.0	2366	38.5	3202	37.2	
35–44	446	18.1	1845	30.0	2291	26.6	
≥ 45	327	13.3	1121	18.2	1448	16.8	<0.001
Education							
None/Primary/Secondary	405	16.5	773	12.6	1178	13.7	
Higher secondary	890	36.2	1805	29.4	2695	31.4	
University	1162	47.3	3561	58.0	4723	54.9	
Place of birth							<0.001
Spain	2271	92.2	5217	84.7	7488	86.9	
Latin-America	117	4.7	544	8.8	661	7.7	
Other country	76	3.1	395	6.4	471	5.5	
Place of residence^b^							0.073
Spain	2310	96.0	5698	95.0	8008	95.2	
Latin-America	23	1.0	91	1.5	114	1.4	
Other country	74	3.1	212	3.5	286	3.4	
City residence (inhabitants)^b^							<0.001
< 100,000	1014	42.2	1875	31.3	2889	34.4	
100,000–500,000	616	25.6	1428	23.8	2044	24.4	
> 500,000	773	32.2	2686	44.8	3459	41.2	
Involvement with the gay scene							<0.001
Member of a gay CBO^c^	59	2.5	391	6.6	450	5.4	
Not member of a gay CBO-Frequents the gay scene	875	37.3	3530	59.6	4405	53.3	
Not related to gay scene	1410	60.2	2006	33.8	3416	41.3	
No. of male sexual partners^b^							<0.001
0	129	5.3	121	2.0	250	2.9	
1–4	1216	49.9	2109	34.7	3325	39.1	
5–9	540	22.2	1316	21.7	1856	21.8	
10–19	341	14.0	1315	21.7	1656	19.5	
≥ 20	210	8.6	1211	19.9	1421	16.7	
Anal intercourse and condom use^b^							<0.001
No anal intercourse	129	5.3	121	2.0	250	3.0	
Had but all were protected	937	38.7	2185	36.2	3122	36.9	
Had unprotected anal sex	1357	56.0	3734	61.8	5091	60.2	
Ever paid for sex	176	7.2	419	6.9	595	7.0	0.572
Ever been paid for sex	99	4.1	286	4.7	385	4.5	0.201
Injected drug use (Ever)	27	1.1	72	1.2	99	1.2	0.763
No. of previous HIV tests							
1			1302	21.2			
2			1320	21.4			
> 2			3534	57.4			
Time since last HIV test (years)							
< 1			2747	44.7			
1–3			2527	41.1			
4–5			451	7.3			
6–10			307	5.0			
> 10			116	1.9			
HIV testing intention in the next year							<0.001
Certainly yes	258	10.5	2793	45.4	3051	35.4	
Probably yes	434	17.6	1544	25.1	1978	23.0	
Not sure	984	40.0	1125	18.3	2109	24.5	
Probably no	576	23.4	551	9.0	1127	13.1	
Certainly no	209	8.5	137	2.2	346	4.0	
Knows about the availability of self-testing in the USA							<0.001
I had no idea about it	2076	84.4	4697	76.6	6773	78.8	
I thought it could exist, although I had never seen it	284	11.5	1014	16.5	1298	15.1	
Yes I knew about it	100	4.1	424	6.9	524	6.1	

^a^Level of significance was examined by chi-square test; ^b^Last 12 months; ^c^CBO: Community based organization

Among all the participants, 56.6 % were under 35 years old, 54.9 % had a university degree, only 13.2 % was born abroad, and 41.3 % had no involvement in the gay scene. Some 60.2 % reported unprotected anal intercourse during the last 12 months, 7.0 % had ever paid for sex, and an additional 4.5 % reported having ever been paid for sex. The reported prevalence of injected drug use was very low (1.2 %). Overall, 58.4 % had the intention to be tested for HIV in the next year, although this proportion was lower among those who had never been tested (28.1 %); 6.1 % knew about the availability of the over-the-counter self-test in the U.S. Among those who had ever been tested, 57.4 % had been tested at least three times, and 44.7 % had done so within the last year.

### Knowledge of online sale of unauthorized rapid HIV home kits for self-testing

Overall, only 360 MSM (4.2 %; 95 % CI: 3.8–4.6) knew that they could buy unauthorized test kits online to perform self-testing, and an additional 12.7 % (95 % CI: 12.0–13.4) thought that such kits might exist, although they had never seen them. Sixty four (18.3 %) out of 350 MSM knew a person who had performed an HIV self-test.

The channels of information through which MSM seem to have come across HIV self-testing were: internet commercials (53.1 %), online videos-trailers talking about self-testing (24.5 %), friends (18.9 %), advertisements posted in gay dating sites (16.7 %), announcements in gay associations (12.2 %), and via their sexual partners (4.7 %).

In the univariable logistic regression for rare events analysis (Table 2), the knowledge that unauthorized self-tests for HIV kits can be purchased online was significantly and positively associated with being born and residing the last year in a foreign non-Latin American country (OR: 1.9; 95 % CI: 1.3–2.8 and OR: 2.0; 95 % CI: 1.3–3.1, respectively), living in a city with more than 500,000 inhabitants (OR: 1.3; 95 % CI: 1.1–1.6), having at least two previous HIV tests (OR: 1.9; 95 % CI: 1.5–2.3), having the intention of being tested in the upcoming year (OR: 1.9; 95 % CI: 1.5–2.4), and knowing about the US approval of HIV self-testing (OR: 5.2; 95 % CI: 4.2–6.5). Knowledge of possible online purchase of these kits was negatively associated with not being a member of a gay organization (OR: 0.5; 95 % CI: 0.4–0.8) or having no involvement with the gay scene (OR: 0.5; 95 % CI: 0.4–0.8).

**Table 2:** Knowledge of unauthorized HIV self-testing and associated factors in MSM websites' users. Crude and adjusted analysis

	Number	Percent	Crude odds ratio	95 % confidence interval	Adjusted odds ratio	95 % confidence interval
Age						
< 35	187	3.8	Ref.		Ref.	
≥ 35	171	4.6	1.2	0.9─1.5	1.0	0.8─1.3
Education						
< University	149	3.8	Ref.			
University	210	4.4	1.2	0.9─1.4		
Place of birth						
Spain	296	4.0	Ref.		Ref.	
Latin-America	30	4.5	1.2	0.8─1.7	1.1	0.8─1.7
Other country	34	7.2	1.9	1.3─2.8	1.4	0.9─2.1
Place of residence^b^						
Spain	315	3.9	Ref.		Ref.	
Latin-America	2	1.8	0.6	0.1─2.3	0.4	0.1─1.4
Other country	21	7.3	2.0	1.3─3.1	1.9	1.1─3.1
City of residence (inhabitants)^b^						
≤ 500,000	176	3.6	Ref.		Ref.	
> 500,000	161	4.7	1.3	1.1─1.6	1.1	0.9─1.4
Involvement with the gay scene						
Member of a gay CBO^c^	32	7.1	Ref.		Ref.	
Not member of a gay CBO-Frequents the gay scene	172	3.9	0.5	0.4─0.8	0.7	0.5─1.1
Not related to gay scene	133	3.9	0.5	0.4─0.8	0.9	0.6─1.4
No. of male sexual partners^b^						
0–1	50	3.6	Ref.			
2–4	85	3.9	1.1	0.8─1.6		
5–9	71	3.8	1.1	0.7─1.6		
> 10	138	4.5	1.3	0.9─1.8		
Anal intercourse and condom use^b^						
No anal intercourse	5	2.0	Ref.			
Had anal intercourse (with or without condom use)	335	4.1	1.9	0.8─4.6		
Ever paid for sex						
No	315	4.0	Ref.			
Yes	31	5.2	1.4	0.9─2.0		
Ever been paid for sex						
No	326	4.0	Ref.			
Yes	21	5.5	1.4	0.9─2.2		
Injected drug use						
Ever	3	3.0	Ref.			
Never	338	4.1	1.6	0.4─3.7		
No. of previous HIV tests						
0–1	108	2.9	Ref.		Ref.	
≥ 2	252	5.2	1.9	1.5─2.3	1.4	1.0─1.8
HIV testing intention in the next year						
Certainly no/Probably no/Not sure	99	2.8	Ref.		Ref.	
Certainly yes/Probably yes	259	5.2	1.9	1.5─2.4	1.5	1.2─2.0
Knows about the availability of self-testing in the USA						
I thought it could exist, although I had never seen it/I had no idea about it	158	2.3	Ref.		Ref.	
Yes I knew about it	202	11.1	5.2	4.2─6.5	4.9	3.9─6.2

^a^The level of significance was examined with the rare event logit regression

^b^Last 12 months

^c^CBO: Community based organization

In the multivariable analysis, this knowledge remained significantly associated with having resided in a foreign non-Latin American country in the last year (OR: 1.9; 95 % CI: 1.1–3.1), having at least two previous HIV tests (OR: 1.4; 95 % CI: 1.0–1.8), the intention of being tested in the upcoming year (OR: 1.5; 95 % CI: 1.2–2.0) and being aware that HIV self-testing had become available in the U.S. (OR: 4.9; 95 % CI: 3.9–6.2).

### Self-testing for HIV with an unauthorized rapid kit purchased on the internet

A total of 61 participants (0.7 %; 95 % CI: 0.54–0.91) had ordered from the internet and used the rapid self-test at least once (16.9 % of the 360 MSM who knew about the availability of online unauthorized self-testing kits). Eighty-six point six percent of the HIV kits purchased were blood-based, whereas the percentage of participants that reported having purchased either a saliva or a urine based kit was of 6.7 % in both cases.. Except for one, all self-testers reported that the instructions on how to obtain the sample and perform the self-test were “very easy” (62.7 %) or “quite easy” (35.6 %). All self-testers interpreted a negative test result and all said that instructions explaining how to interpret the self-test were “very easy” (68.4 %) or “quite easy” (31.6 %). Forty-five men performed the test completely alone; eleven were accompanied by their sexual partner and three by a friend. Sixteen of them followed the self-test with a conventional test, which was found to be negative. Eleven of them took the conventional test within the first month and three at a later period.

The unadjusted analysis (Table 3) showed that self-testers were more likely to be over 34 years old (OR: 2.0; 95 % CI: 1.2–3.4), hold a university degree (OR: 1.9; 95 % CI: 1.1–3.4), reside outside Spain in the last 12 months (OR: 3.4; 95 % CI: 1.6–7.3), have at least two previous HIV tests (OR: 2.3; 95 % CI: 1.3–4.2), have the intention to be tested in the upcoming year (OR: 2.6; 95 % CI: 1.4–4.8), and to know that HIV self-testing had become available in U.S. (OR: 5.1; 95 % CI: 3.0–8.4).

**Table 3:** Use of unauthorized HIV self-testing kits and associated factors in MSM websites' users. Crude and adjusted analysis

	Number	Percent	Crude odds ratio	95 % confidence interval	Adjusted odds ratio	95 % confidence interval
Age						
< 35	24	0.5	Ref.		Ref.	
≥ 35	37	1.0	2.0	1.2─3.4	1.7	1.0─2.9
Education						
< University	18	0.5	Ref.		Ref.	
University	43	0.9	1.9	1.1─3.4	1.5	0.8─2.6
Place of birth						
Spain	51	0.7	Ref.			
Latin-America	4	0.6	1.0	0.4─2.8		
Other country	6	1.3	2.0	0.9─4.7		
Place of residence^b^						
Spain	49	0.6	Ref.		Ref.	
Other country	8	2.0	3.4	1.6─7.3	3.1	1.5─6.8
City of residence (inhabitants)^b^						
> 500,000	22	0.6	Ref.			
100,000–500,000	12	0.6	0.9	0.5─1.9		
< 100,000	22	0.8	1.2	0.7─2.2		
Involvement with the gay scene						
Member of a gay CBO^c^	1	0.2	Ref.			
Not member of a gay CBO-Frequents the gay scene	33	0.8	2.1	0.3─15.3		
Not related to gay scene	23	0.7	1.9	0.3─14.0		
No. of male sexual partners^b^						
0–1	8	0.6	Ref.			
2–4	14	0.6	1.1	0.5─2.6		
5–9	12	0.7	1.1	0.5─2.7		
> 10	24	0.8	1.3	0.6─2.9		
Anal intercourse and condom use^b^						
No anal intercourse	1	0.4	Ref.			
Had anal intercourse (with or without condom use)	56	0.7	1.1	0.2─7.6		
Ever paid for sex						
Yes	56	0.7	Ref.			
No	3	0.5	1.2	0.4─3.8		
Ever been paid for sex						
No	3	0.8	Ref.			
Yes	56	0.7	1.3	0.4─4.3		
No. of previous HIV tests						
0–1	15	0.4	Ref.		Ref.	
≥ 2	46	0.9	2.3	1.3─4.2	1.3	0.7─2.5
HIV testing intention in the next year						
Certainly no/Probably no/Not sure	13	0.4	Ref.		Ref.	
Certainly yes/Probably yes	48	1.0	2.6	1.4─4.8	1.8	1.0─3.5
Knows about the availability of self-testing in the USA						
I thought it could exist, although I had never seen it/I had no idea about it	26	0.4	Ref.		Ref.	
Yes I knew about it	35	1.9	5.1	3.0─8.4	4.4	2.6─7.4

^a^The level of significance was examined with the rare event logit regression

^b^Last 12 months

^c^CBO: Community based organization

In the adjusted analysis, being an HIV self-tester was significantly associated with being over 34 years of age (OR: 1.7; 95 % CI: 1.0–2.9), having lived in a country other than Spain the last 12 months (OR: 3.1; 95 % CI: 1.5–6.8), having the intention to test for HIV in the upcoming year (OR: 1.8; 95 % CI: 1.0–3.5) and knowing about the approval of self-testing in the U.S. (OR: 4.4; 95 % CI: 2.6–7.4).

## Discussion

This study shows that less than 5% of Spanish MSM were aware of the availability on the internet of unauthorized and unreliable self-testing kits for HIV, and less than 1% had ever used these kits shortly after having been exposed to mass media information related with the approval of over-the-counter HIV self-testing in the U.S. Knowledge of online availability was associated with residing in a foreign non-Latin American country, having at least two previous HIV tests, intending to be tested for HIV in the next year, and knowing about US approval of self-testing. Ever-use of an HIV self-test was associated with being over 34 years of age, living outside Spain the last 12 months, and knowing about the U.S. approval of self-testing.

The proportion of participants that knew about the availability of unauthorized HIV self-testing online (4.2 %) and the proportion of self-testers (0.7 %) in this study were lower than those observed among Spanish MSM in a previous study carried out between 2010 and 2012 (7.4 and 0.9 % respectively) [10], which was completed 3 months before the U.S. approval of self-testing. Part of this difference could be explained by the fact that all the participants in the previous Spanish study were MSM who sought an HIV test, whereas three out of ten participants in the present study had never been tested. The number of HIV tests was associated with knowledge of online availability of HIV testing in both studies, thus, the fact that almost one-third of the recruited sample had no HIV testing experience could partially explain the slightly lower knowledge found in this study. If we re-run our analysis excluding those participants who reported no previous testing experience, the prevalence of knowledge and use both increase slightly (4.8 % and 1.0 % respectively). Thus, the prevalence of knowledge still remains lower than that of the Spanish study where all men had previous HIV testing history [10] whereas the prevalence of participants who reported ever having bought an HIV self-testing kit is slightly higher. However, it is also true that participants in the present study were recruited online, and it is likely that they are more familiar with the internet.

Knowledge and use of HIV self-testing in the two Spanish studies were substantially lower than in a French study carried out among MSM in 2009 using a web-based recruitment approach very similar to the one used in the present study [15]. In our study, knowledge of the existence of unauthorized kits was associated with living in a non-Latin American country, whereas their use was found to be associated with having lived outside of Spain during the last 12 months. Thus, even though HIV self-testing has proven to be an acceptable and convenient HIV testing method in the U.S. and other high income countries [16], we can conclude that there is no evidence that the information related with the approval of over-the-counter self-test kits in the U.S. has increased the awareness of the existence of an online market of self-test kits in MSM in Spain. Finally, we should bear in mind some limitations arising from the design and procedures of the study: an online recruitment of a convenience sample. Although this procedure allowed us to reach a large sample of electronically-connected key populations – with participation from all of Spain’s 17 autonomous regions -- generalization to the total Spanish MSM population should be made with caution. The percentage of non-response to questions on self-testing (8.6 %) may have introduced a response bias. The non-respondents were significantly different in some characteristics from those who did answer questions related to self-testing: they tended to be older, have lower educational level, and a slightly higher presence of Latin Americans, and also of participants who were not thinking of getting tested in the next year and who had not heard of the availability of self-testing in the U.S.

As no identification data was collected, some individuals could have submitted more than one questionnaire. To control this possibility, participants were shown a note before being directed to the first page of the questionnaire asking them not to fill it out if they had already participated in the study. We also checked for duplicate entries during quality control analysis as a way of reducing the effect of this limitation. Because the questionnaire was self-completed rather than administered by an interviewer, it was easier to stop before answering all the questions, but this also allowed respondents to answer in a more relaxed environment less prone to social desirability bias. Finally, despite having employed a special methodology to analyse the correlates of the main outcomes, the variability caused by the low number of events could have limited the robustness of the estimates.

## Conclusions

In conclusion, knowledge of HIV status and access to self-testing remains low This situation could change quickly, however, considering that an HIV-self test is already sold legally in the UK [8] and France [9]. Some Spanish MSM may well decide to purchase and use the legal and reliable commercial kits sold in these two countries, considering the fluid interconnections among gay communities in these countries and the absence of borders for goods and merchandise. In countries where self-testing is yet to be authorized, there may also be an increase in the unregulated trade of illegal kits with similar presentations to those approved in the aforementioned countries. Nevertheless, these tests will not necessarily comply with the handling, storing and distribution conditions of legally purchased tests. Furthermore, they will not offer any kind of telephone or web support for interpretation of results, counselling, referral or linkage to services for care and support. Moreover, other presentations with fewer, if any, guarantees, will continue to be sold online and probably at a lower cost. All these considerations should be taken into account when evaluating the advantages and drawbacks of regulating these tests in EU member countries.

## Abbreviations

CBO, community based organization; CI, confidence interval; FDA, United States Food and Drug Administration; LGTB, lesbian gay transexual and bisexual; MSM, men who has sex with men; NGO, non-governmental organization; OR, odds ratio; STI, sexually transmitted infections; UK, United Kingdom

## Additional file

Additional file 1: Contains the data used in the analysis. (XLSX 9721 kb)
